# Factors influencing the uptake of family planning services in the Talensi District, Ghana

**DOI:** 10.11604/pamj.2015.20.10.5301

**Published:** 2015-01-05

**Authors:** Paschal Awingura Apanga, Matthew Ayamba Adam

**Affiliations:** 1 Ghana Health Service, Talensi district, Upper East Region, Ghana

**Keywords:** Family planning, contraceptives, uptake, Talensi district, Ghana

## Abstract

**Introduction:**

Usage of family planning services in developing countries have been found to avert unintended pregnancies, reduce maternal and child mortality, however, it's usage still remains low. Hence, the objective of this study was to investigate the factors that influence the decision of women in fertility age to go for family planning services.

**Methods:**

This was a descriptive cross-sectional study conducted in Talensi district in the Upper East Region of Ghana. Systematic random sampling was used to recruit 280 residents aged 15-49 years and data was analysed using SPSS version 21.0.

**Results:**

The study revealed that 89% (249/280), of respondents were aware of family planning services, 18% (50/280) of respondents had used family planning services in the past. Parity and educational level of respondents were positively associated with usage of family planning services (P<0.05). Major motivating factors to the usage of family planning service were to space children, 94% (47/50) and to prevent pregnancy and sexual transmitted infections 84% (42/50). Major reasons for not accessing family planning services were opposition from husbands, 90% (207/230) and misconceptions about family planning, 83% (191/230).

**Conclusion:**

Although most women were aware of family planning services in the Talensi district, the uptake of the service was low. Thus, there is the need for the office of the district health directorate to intensify health education on the benefits of family planning with male involvement. The government should also scale up family planning services in the district to make it more accessible.

## Introduction

Family planning is widely acknowledged as an important intervention towards achieving Millennium Development Goals (MDGs) four (4) and five (5) as it has proven to reduce maternal and child mortality [1–3]. Family planning can prevent unwanted pregnancies and unsafe abortions. Some family planning methods such as condom usage can protect individuals from Sexually Transmitted Infections (STIs) including HIV/AIDS [1–3]. Family planning has also been found to promote gender equality as well as promote educational and economic empowerment for women [4]. Despite the enormous benefits of family planning services, the uptake of the service still remains low in Sub-Saharan Africa [1]. This has resulted into high rates of unwanted pregnancies, unplanned deliveries, unsafe abortions and maternal mortalities in Sub-Saharan Africa of which Ghana is no exception [1, 5]. The low uptake of family planning is largely blamed on many factors. It has been observed that the awareness of the availability of family planning services has a great influence on the uptake of family planning services [6]. Additionally, even though some women are aware of the availability of family planning services, they are not properly informed about the various forms of family planning methods and how they work [7]. Some of the women who went for family planning services were not adequately counselled on the side effects of some of the family planning methods [7]. For example, in Uganda, some women stopped using contraceptives after they experienced what they perceived were side effects of the contraceptives [8].

Although most people are aware of the benefits of family planning services, they complained that it was difficult to access family planning services as such services were provided by health facilities that were far from their homes [9]. In addition, religious inclination has been noted to be a major constrain to the uptake of family planning services in Africa [9, 10]. Also, some individuals perceived that family planning services were meant for only married couples whilst others fear that they will become sexually promiscuous if they go for family planning services once they cannot become pregnant [1, 9, 10]. In Ghana, some efforts have been made by the government of Ghana and non-governmental organizations through the implementation of various programmes to improve the coverage of family planning services in the country [9]. Although some successes have been chucked in the area of awareness of family planning services in the country, the unmet need for family planning still remains high [1, 9]. The Ghana Demographic and Health Survey (GDHS) observed that a large number of women have an unmet need for family planning as the acceptor rate for family planning services remains low [11]. It is on this note the Ghana Health Service argues that the lives of mothers and children will be improved and maternal mortality reduced if family planning acceptor rate is improved [1, 11].

Similarly, the Talensi district in Upper East Region (UER) of Ghana is not spared from this predicament as the acceptor rate for family planning services also remains low. The Talensi district offers free family planning services to clients in most of the health facilities in the district [12]. Despite the provision of free family planning services, the district reported 19% family planning acceptor rate in 2013, which is currently below the Ghana Health Service national family planning acceptor target rate of 23.3% [12]. The regional annual health report showed an increase in teenage pregnancies as well as unsafe abortions in the Talensi district [12]. Three maternal deaths were recorded in the Talensi district in 2011 as a result of unsafe abortions [12]. The increase in teenage pregnancies and unsafe abortions as well as the maternal mortalities that occurred could have been prevented if uptake of family planning services were improved. The decision to investigate the factors that influence the uptake of family planning services in the Talensi district is imperative as very little is known about the factors that influence the decision of people to go for family planning services in the Talensi district.

## Methods

### Settings, population and study design

This was a cross-sectional quantitative survey which was conducted between January and May, 2014. Questionnaires were administered to women aged 15-49years in households in the eight (8) sub-districts of the Talensi district. The Talensi district is one of thirteen districts in the Upper East Region of Ghana. The district has a total of population of 84,712 with a population of 19,738 of the women in fertility age (15-49years) with most inhabitants of the district being peasant farmers [12]. The district has eighteen (18) health care facilities which comprise of health centres, clinics and Community-based Health Planning and Services (CHPS) compounds of which sixteen (16) of the facilities offer free family planning services. Some of the family planning methods that are currently being offered include; Jadelle, Norplant, Condoms, Depo-provera, combined oral contraceptive pills etc [12].

### Sample size

A sample size of 280 was derived using sample size formula for a single population. The assumptions made were using a 95% confidence interval, 5% margin of error and 23.3% national expected proportion of uptake of family planning service among Women In Fertility Age (WIFA).

n= Z^2^P (1-P)/(d)^2^

Where n is the required sample size, P= 23.3% (0.233), Z= 1.96 and d= 5% (0.05). n= (1.96)^2^ (0.233 × 0.767)/(0.05)^2^ =275 which is 280 (to the nearest tenth).

### Sampling method

A total of 280 of women aged 15-49years were recruited for the study using systematic random sampling technique. The 280 participants recruited were from households in the 8 sub-districts that make up the Talensi district (35 households from each sub-district) with one participant from each household. The 280 participants were recruited by the researchers themselves with the help of a community volunteer from each sub-district. The community volunteer helped researchers to approach community leaders to inform them about the purpose of the study and to obtain permission from them to recruit and interview potential participants as this is standard community entry protocol for conducting research within local community context in Ghana [13]. The presence of community volunteers in the team may have influenced the high response rate of 100% as they are highly respected with good knowledge of local residents. All households were numbered and a sampling interval, n=5 was used to select the first household. Subsequent selection of every 5th household then followed in same direction. Using this technique about 284 households was selected but 4 households were dropped because no one was available at the house to be recruited at the time of the survey.

### Measurement

The questionnaires were structured with closed ended pre-coded questions and administered to participants by the Researchers. The questionnaires were divided into four parts. The first part was the socio-demographics whilst the second part was the awareness and usage of family planning services. The third part of the questionnaire provided various reasons that motivated women to access family planning services and the fourth part provided various reasons that discouraged women from using the service. The questionnaires were administered in a language that participants were comfortable to respond. The questionnaires were first piloted on ten (10) participants in the Nabdam district, a neighbouring district which shares border with the Talensi district. Piloting of the questions was done to ensure that the questions were more refined for participants to respond without difficulties [14].

### Data analysis

All data were entered into SPSS version 21.0 and analyzed. The P-value of 0.05 was taken for statistical significance. The association between the binary outcome, usage of family planning services (yes or no) with independent categorical variables such as marital status, age, religion, parity and educational level were investigated using chi-squared test. Binary logistic regression model with the outcome, those who had used family planning services was used to investigate the influence of independent variables such marital status, age, religion, parity and educational level on the uptake of family planning services. Descriptive summary statistics was also used to report various reasons that motivated respondents to go for family planning services as well as reasons why some respondents did not use the service in the district.

### Ethics issues

This study was approved by the Ethics committee board of the Catholic University college of Ghana and the Talensi district health directorate. Written consent was obtained from participants and for participants who were within the ages of 15-17years written consent was obtained from their guardians on behalf of them. All participants who consented to take part in the survey were well informed about the purpose of the study and why they were being asked to participate. They were also informed that participation was voluntary and they had the right to withdraw from the study even after they had participated.

## Results

Table 1 shows socio-demographic characteristics of 280 participants in relation to utilization of family planning services who were recruited for the survey. Most of the respondents in the study were within the age group of 15-20 years, 25% (70/280). Of the 280 respondents, majority were married, 73% (204/280) and 20% (56/280) of respondents were single. Most of the respondents were Christians, 56% (157/280). With regards to education, most of the respondents in the study had a senior high school level of education, 24 (67/280) and respondents who had four or more children, 55% (154/280) were the majority in the study. Table 1 also shows the results of the association between socio-demographic characteristics and the binary outcome, having used family planning services (yes or no) using chi-squared test. It showed that educational level and parity of respondents were positively associated with usage of family planning services (p<0.05) whereas marital status, age group and religion were not statistically significant.

**Table 1:** Utilization of family planning services in relation to socio-demographic characteristics (n=280)

Variable	Utilization of family planning services						P-value
	Yes	%	No	%	n	%	
**Marital status**							0.061#
Married	16	8	188	92	204	73	
Single	34	61	22	39	56	20	
Widowed	0	0	20	100	20	7	
Divorced	0	0	0	0	0	0	
Total	50		230		280	100	
**Age group**							0.524*
15-20	25	36	45	64	70	25	
21-26	5	7	62	93	67	24	
27-32	14	21	53	79	67	24	
33-38	3	7	42	93	45	16	
39-49	3	10	28	90	31	11	
Total	50		230		280	100	
**Religion**							0.624#
Christian	19		138		157	56	
Moslem	14		53		67	24	
Traditional	17		39		56	20	
Total	50		230		280	100	
**Educational level**							0.027*
Tertiary	28	4	34	96	64	23	
Senior high school	11	16	56	84	67	24	
Junior high school	6	11	47	89	53	19	
Primary	3	5	56	95	59	21	
None	2	3	37	95	39	13	
Total	50		230		280	100	
**Parity**							0.012*
4 or more children	24	16	130	84	154	55	
1 -3 children	16	20	64	80	80	29	
No child	10	22	36	78	46	16	
Total	50		230		280	100	

n= frequency %= percentage

* =P-value from chi-squared test for trend or linear by linear association.

# = P-value from Pearson's chi-squared test

Table 2 shows a further logistic regression model that have demonstrated that educated women are more likely to use family planning services as compared their peers who did not receive formal education (P=0.01). Similarly, the higher the parity of women the more likely they are to use the service as compared to people with lower parity (p=0.03). However, the association between age, marital status, religion and usage of family planning services were not statistically significant (P>0.05).

**Table 2:** Logistic registration of demographic factors influencing family planning services utilization n=280)

Variables	OR	95%CI	P
Age	0.024	(0.231;1.658)	0.21
Marital status	0.512	(0.172; 1.342)	0.12
Religion	0.493	(0.169;1.239)	0.32
Parity	1.312	(0.863; 1.785)	0.03
Education	1.079	(0.367; 1.823)	0.01

OR =Odd Ratios, CI =Confident Interval and P=probability value

Table 3 stipulates various responses by study participants towards the usage of family planning services. Of the few respondents who had accessed family planning services in the past, 18% (50/280) provided various reasons that lead to the decision to undertake family planning services. Major reasons that influenced the decision of women to go for family planning services were to space children, 94% (47/50) and to prevent pregnancy and Sexually Transmitted Infections (STIs), 84% (42/50). Majority of survey respondents who had not accessed family planning services, 82% (230/280) also cited various reasons for not using the service. Opposition from husbands for their wives not to access the service, 90% (207/230) as well as misconceptions about family planning methods, 83% (191/230) were reported as major reasons against usage of family services.

**Table 3:** Responses to usage of family planning services

**Reasons for accessing family planning services**	**n (50)**	**%**
To space children	47	94
To have sex without children	6	12
Fear of pregnancy	22	44
To prevent pregnancy and STIs	42	84
**Reasons for not accessing family planning services**	**n (230)**	**%**
Against my religious faith	69	30
Fear of sexual promiscuity	14	6
Opposition from husbands	207	90
Distance to accessing the service	28	12
Poor health staff attitude	18	8
Side effects	32	14
Misconceptions	191	83

n=frequency %=percentage NB: Some respondents gave more than one reason

## Discussion

The study was a cross-sectional study that investigated the factors that influence the utilization of family planning services among women within the age group of 15-49 years in the Talensi district. The findings suggest that although majority of the women were generally aware of family planning services in the district, usage of the service was low. Major reasons cited for not using service included husbands opposition against their wives using family planning services, this argument is in line with the observation made by Allen et al. (2014) in Uganda [15]. This is a major constrain as women in Ghana cannot take decisions for themselves without the approval of their husbands, who are regarded as the head of the family. Therefore it makes women more unlikely to use contraceptives if their husbands disagree. Another major reason that was reported were some perceived misconceptions about family planning services. Some of these misconceptions included respondents who did not go for family planning services because they perceived that it was meant for only married people whilst others perceived that contraceptives were harmful to the womb. These findings were consistent with studies reported by Meka et al. (2013) in Nigeria and Gebremariam and Addissie (2014) in Ethiopia respectively [16, 17]. Other reasons that were reported for using family planning services in the district were; spacing of children and the desire of some women to prevent pregnancy and Sexual Transmitted Infections (STIs). These reasons were also found in similar studies by Abdurahman et al. (2014) and Awusabo-Asare et al. (2006) respectively [18, 19].

These current findings have demonstrated that although the government has created an appreciable level of awareness about family planning services through its educational campaign programmes, only 18% of respondents have used the service. Hence, there is the need for the Ghana Health Service to re-visit the methods currently adopted to deliver family planning messages in rural communities in the Talensi district. Family planning messages should be integrated into existing health education programmes as it could help increase awareness, access and utilisation. Also, family planning educational messages should focus on the involvement of male partners in delivering the service as well as the benefits of family planning services as it will help reduce misconceptions about family planning services. On the other hand, the high awareness of family planning services coupled with low usage of the service compares favourably with Hamid and Stephenson (2006) study in Pakistan which observed that despite the high awareness of family planning services in the community, very few people used the service [20]. However, the high awareness of family planning services in the Talensi district may be attributed to the Community-based health planning and services (CHPS) compounds concept that was introduced to make health care more accessible to the rural communities as well as empowering them to have greater control of their own health [21, 22].

In this study, it was found that the educational level of respondents was positively associated with utilization of family planning services. This finding suggests that people with higher education are more likely to go for family planning services than their counterparts with lower education. This agrees with other studies that revealed that women with higher education tends to be better informed about family planning services and are more likely to use the service than their peers with lower education [9, 23–25]. Parity was also positively associated with usage of family planning services. This conforms to similar studies reported in Nigeria where women are more likely to use family planning service if they have three or more children [26]. Findings of this study are very useful as it can inform policy and decision making in the Ghana Health Service to help increase the family planning acceptor rate in Talensi district and the Upper East Region as a whole. However, some weakness in the study design limits the generalisation of findings to entire population of Talensi district as participants were conveniently selected from households rather than adopting a random sampling approach, which may not be representative of the general population. However, we ensured that participants were recruited randomly from households to address any problem of selection bias. Also, husbands should be involved in further research to investigate their perceptions about usage of family planning services.

## Conclusion

In conclusion, this study has provided evidence to demonstrate that although the awareness of family planning services among community members could be high, it does not necessarily increase the uptake of family planning services if community members are not well informed about the benefits of family planning. It is therefore essential to organise educational campaigns on the awareness of family planning services by emphasising on the benefits of the services as it will help reduce misconceptions, and increase access and utilization of family planning services. Males should also be educated on the benefits of family planning services so as to reduce opposition from husbands. Furthermore, if women have the desire to space their children as well as prevent pregnancy and STIs, they are more likely to use the service.

## References

[1] Eliason S, Baiden F, Quansah-Asare G, Graham-Hayfron Y, Bonsu D, Phillips J, Awusabo-Asare K (2013). Factors influencing the intention of women in rural Ghana to adopt postpartum family planning. Reprod Health [Online].

[2] Cates WJ, Abdool Karim Q, El-Sadr W, Haffner DW, Kalema-Zikusoka G (2010). Global development. Family planning and the millennium development goals. Science..

[3] Sachs JD, McArthur JW (2005). The millennium project: a plan for meeting the millennium development goals. Lancet..

[4] Yue K, O'Donnel C, Sparks PL (2010). The effect of spousal communication on contraceptive use in Central Terai, Nepal. Patient Educ Couns..

[5] Crossette B (2005). Reproductive health and the millennium development goals: the missing link. Stud Fam Plann..

[6] Lauria L, Donati S, Spinelli A, Bonciani M, Grandolfo ME (2014). The effect of contraceptive counselling in the pre and post-natal period on contraceptive use at three months after delivery among Italian and immigrant women. Ann Ist Super Sanita..

[7] Malini B, Narayanan E (2014). Unmet need for family planning among married women of reproductive age group in urban Tamil Nadu. Journal of Family & Community Medicine..

[8] Kabagenyi A, Jennings L, Reid A, Nalwadda G, Ntozi J, Atuyambe L (2014). Barriers to male involvement in contraceptive uptake and reproductive health services: a qualitative study of men and women's perceptions in two rural districts in Uganda. Reprod Health..

[9] Gaetano M, Lutuf A, Zaake D, Annika J (2014). Predictors of Contraceptive use Among Female Adolescents in Ghana. Afr J Reprod Health March..

[10] Odimegwu C (2005). Influence of religion on adolescent sexual attitudes and behaviour among Nigerian University students: Affiliation or commitment?. Afr J Reprod Health.

[11] Ghana Statistical Service (GSS), Ghana Health Service (GHS), ICF Macro (2009). Ghana Demographic and Health Survey 2008: Key Findings.

[12] Ghana Health Service (2012).

[13] Hill Z, Manu A, Tawiah-Agyemang C, Gyan T, Turner K, Weobong B (2008). How did formative research inform the development of a home-based neonatal care intervention in rural Ghana?. J perinatol [Online].

[14] Saunders MNK, Lewis P, Thornhill A (2003). Research methods for business students.

[15] Allen K, Larissa J, Alice R, Gorette N, James N, Lynn A (2014). Barriers to male involvement in contraceptiveuptake and reproductive health services: a qualitative study of men and women's perceptions in two rural districts in Uganda. Reprod Health [Online].

[16] Meka A, Okwara EC, Meka AO (2013). Contraception among bankers in an urban community in Lagos State, Nigeria. Pan Afr Med J..

[17] Gebremariam A, Addissie A (2014). Intention to use long acting and permanent contraceptive methods and factors affecting it among married women in Adigrat town, Tigray, Northern Ethiopia. Reprod Health.

[18] Awusabo-Asare K, Biddlecom A, Kumi-Kyereme A, Patterson K (2006). Adolescent Sexual and Reproductive Health in Ghana.

[19] Abdurahman M, Desalegn W, Amsalu F, Berihun M (2014). Determinants of modern contraceptive utilization among married women of reproductive age groupin North Shoa Zone, Amhara Region, Ethiopia. Reprod Health [Online].

[20] Hamid S, Stephenson R (2006). Provider and Health Facility Influences on Contraceptive Adoption in Urban Pakistan. Int Fam Plan Perspect..

[21] Nyonator FK (2005). The Ghana community-based health Planning and Service Initiative for scaling up service delivery innovation. Health Policy and Practice Journal..

[22] Baatiema L, Skovdal M, Rifkin S, Campbell C (2013). Assessing participation in a community-based health planning and services programme in Ghana. BMC Health Serv Res..

[23] Weller S, Davis K (2002). Condom effectiveness in reducing heterosexual HIV transmission. Cochrane Database Syst Rev..

[24] Uwaezuoke IOA, Uzochukwu BSC, Nwagbo DFE, Onwujekwe OE (2004). Determinants of teenage pregnancy in rural communities of Abia State, South East Nigeria. Journal of College of Medicine..

[25] Karigu K, Zabin LS (1995). Contraceptive use among high school students in Kenya. Int Fam Plan Perspect..

[26] Anate M (1995). Factors influencing family planning use in Ilorin, Nigeria. East Afr Med J..

